# Seed Production and Pollinator Dependence in Native Wildflowers: Guiding Species Selection for Conservation Plantings

**DOI:** 10.1002/ece3.72127

**Published:** 2025-09-22

**Authors:** Anthony P. Abbate, Joshua W. Campbell, Anthony W. Cuminale, Natalie M. West, Geoffrey R. Williams

**Affiliations:** ^1^ Bee Center, Department of Entomology and Plant Pathology Auburn University Auburn Alabama USA; ^2^ USDA—Agricultural Research Service, Pest Management Research Unit Northern Plains Agricultural Research Laboratory Sidney Montana USA

**Keywords:** insect exclusion, native pollinators, pollination, seeds, wildflowers

## Abstract

The diversity of plants and their animal pollinators is in decline due to anthropogenic pressures such as habitat loss, pesticide use, and agricultural intensification. Approximately 78% of angiosperms rely on animal‐mediated pollination, yet data on the pollination biology of many native wildflower species is lacking. We investigated the pollination biology of eight wildflower species native to the southeastern United States, including *Bidens laevis, Desmanthus illinoensis, Echinacea purpurea, Eryngium yuccifolium, Gaillardia pulchella, Monarda punctata, Verbesina alternifolia,* and *Verbesina virginica,* which are either recommended by the USDA—Natural Resource Conservation Service or the Xerces Society to enhance native pollinator habitat. We evaluated each species' dependence on insect pollinators through an insect exclusion experiment and documented insect floral visitors via sweep netting surveys followed by a plant‐pollinator network analysis. For most species, open‐pollinated inflorescences produced significantly more and/or heavier seeds per inflorescence compared to those excluded from insect visitors, indicating a high degree of pollinator influence. In comparison, several species exhibited potential for self‐pollination. A total of 1417 insects, representing 73 genera/species from 21 families and 4 orders, were observed visiting the wildflower species. These findings indicate the importance of insect pollinators for maximizing seed production and quality in these native wildflower species and underscore the importance of considering the pollination requirements when selecting species for conservation plantings. Our results highlight that some native wildflower species reproduce and set seed in the absence of pollinators, making them valuable for providing floral resources in degraded habitats, while strongly pollinator‐dependent species may be better suited for conservation plantings in areas with robust or recovering pollinator communities.

## Graphical abstract

We investigated the pollination biology of eight native wildflower species in the southeastern U.S. to assess their dependence on insect pollinators and potential for self‐pollination. Most species showed significantly higher seed production and weight when open‐pollinated, indicating strong pollinator dependence, though some could reproduce without insect visitors. These results highlight the need to consider pollination requirements when selecting species for conservation plantings, with some suited for degraded habitats and others for areas with robust pollinator communities.

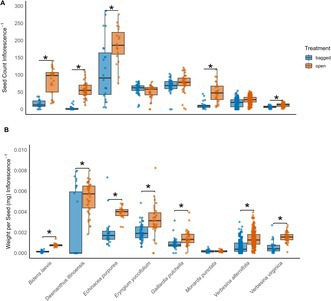

## 1 Introduction

Within the United States, the pollination biology of rare and crop plant species has been evaluated to design effective management plans (Boyd 1994; Dute et al. 2004; Isaacs and Kirk 2010; Garratt et al. 2014; Paris and Boyd 2018). Approximately 78% of all plant species rely on animal‐mediated pollination, whereas others rely on abiotic pollen vectors such as wind or are predominantly self‐pollinating (Ollerton et al. 2011; Wright et al. 2013). Despite our knowledge of the pollination mechanisms of rare and crop plant species, the pollination biology of most wildflower species remains largely unknown. The provision of sufficient forage in the form of pollen and nectar is a key element in supporting and promoting pollinators within human‐impacted landscapes (Williams et al. 2015). However, the reciprocal benefits to wildflower populations in the landscape have seldom been quantified. Recent government programs in the United States and abroad have promoted the planting of native wildflowers to benefit insect pollinators, yet basic ecological information such as the plants' pollination biology and the common insect pollinators that visit them for pollen and/or nectar rewards is often lacking. Plant characteristics such as floral morphology, bloom period, and floral rewards attract and reward different insect pollinator assemblages that aid in the production of viable seeds and plant population persistence (Erickson, Grozinger, and Patch 2022; Erickson, Junker, et al. 2022). Pollinator‐dependent wildflower species without adequate pollinator availability will both fail to persist in the environment and to provide future populations of insect pollinators with the floral resources that are crucial for their development, maintenance, and reproduction (Payne et al. 1989; Khan and Chaudory 1995; Seeley 2009; Stein et al. 2017; Leach and Drummond 2018). Furthermore, understanding the pollination biology of native wildflowers commonly utilized in seed mixes is important for the development of conservation management plans to conserve wildflower diversity and support the insect pollinators that rely on them.

Plants benefit both directly and indirectly from insect pollination. For most flowering plant species, there is a direct link between pollinator dependency and the number of mature seeds produced. For example, the more pollinator visits to a flower, the more pollen is deposited, resulting in the production of a greater number of seeds (Snow 1982; Boyd 1994; Steffan‐Dewenter et al. 2001; Oz et al. 2009; Bommarco et al. 2012; Campbell et al. 2018; Stavert et al. 2020). Insect pollinators can increase pollen delivery and improve reproductive success, even in plant species with self‐ or mixed self‐outcrossing breeding systems (e.g., Saunders 2018; Abbate et al. 2023). Additional pollinator visits often lead to increased seed or fruit set by improving both within and between flower pollen transfer (Moreira‐Hernández and Muchhala 2019). Yet this link may be indirectly strengthened by how plant‐pollinator interactions impact the quality of the seeds produced (Aizen and Harder 2007). Insects carrying pollen among multiple flowers can promote genetic diversity and outcrossing, which can increase seed weight as well as seed production (Blaauw and Isaacs 2014; Moreira‐Hernández and Muchhala 2019). The number and size of seeds or fruits produced are often dependent on the quantity and quality of deposited pollen grains (Björkman 1995; Dogterom et al. 2000). Furthermore, seed weights of pollinator‐dependent plants are often reduced when insect pollinators are excluded from inflorescences (Atmowidi et al. 2007; Oz et al. 2009; Bommarco et al. 2012), and seeds with reduced weights are less fit compared to larger seeds produced by the same plant species (Banovetz and Scheiner 1994). If planted species benefit from direct or indirect interactions with pollinators, then conservation plantings are more likely to provide a consistent resource for insect pollinators, increasing the overall conservation value of planted areas.

Thoughtful plant selection is therefore essential in conservation efforts. Including species that are self‐pollinated or partially self‐pollinated can be valuable in degraded habitats that lack robust insect pollinator communities. Self‐pollinated plants can establish and provide floral resources even in the absence of consistent pollination services (Makowski et al. 2024), though they may still benefit from pollinator interactions. Conversely, in areas experiencing intact or recovering insect pollinator populations, incorporating pollinator‐dependent plant species can further enhance ecosystem function by reinforcing mutualistic interactions and increasing redundancy, ultimately strengthening plant‐pollinator interactions. Creating wildflower mixes that support both generalist and specialist pollinators in both degraded and restored sites should be a focal point.

The primary purpose of this study was to (i) assess the pollination requirements of commonly planted wildflower species through an insect exclusion study, (ii) document and compare insect floral visitor abundance and richness among wildflower species through targeted sweep netting surveys, and (iii) construct a plant‐pollinator network to highlight interactions between insect floral visitors and native wildflower species. Because the selected species are planted to enhance pollinator habitat and to support insect pollinators, we expected that wildflowers would be visited by an abundance and diversity of insect pollinators and hypothesized that insect visitation to flowers would increase seed production and seed weights (a common metric of seed quality), compared to flowers excluded from insect pollinators (Steffan‐Dewenter and Tscharntke 1999; Albrecht et al. 2012). Alternatively, wildflower species that have similar seed production regardless of insect visitation would be less dependent upon insect pollinators and potentially host fewer floral visitors.

## 2 Materials and Methods

### 2.1 Study Design

We assessed the pollination requirements of nine species of wildflowers native to the southeastern United States that are either recommended by the USDA—Natural Resources Conservation Service (USDA‐NRCS): *Desmanthus illinoensis* (Michx.) MacMill. Ex. B.L. Rob. & Fernald, *Desmodium floridanum* Chapm., *Echinacea purpurea* (L.) Moench, *Eryngium yuccifolium* Michx., *Gaillardia pulchella* Foug., *Monarda punctata* L., *Verbesina alternifolia* (L.) Britton ex Kearney, and *Verbesina virginica* L, and/or according to the Xerces Society, are thought to have high pollinator value: *Bidens laevis* (L.) Britton, Sterns & Poggenb, *E. purpurea* , *E. yuccifolium* , *G. pulchella, M* *. punctata* , *V. alternifolia*, and *V. virginica* (Xerces Society 2023). These nine species are commonly used to enhance native pollinator habitat and are recommended for use within pollinator plantings to support insect pollinators, but their pollination biologies, including seed production and reliance upon insect pollinators to set fruit, are not fully understood. More specifically, *D. illinoensis* is widely used in revegetation projects, is a host for several skipper butterflies, and has been presumed to be self‐pollinating (Latting 1961; USDA‐Natural Resources Conservation 2012). *Desmodium floridanum* is recommended by the USDA‐NRCS Conservation Stewardship Program (CSP) to support pollinators and beneficial insects, is a host plant for the velvet bean caterpillar ( *Anticarsia gemmatalis* Hübner, 1818: Erebidae), and lacks documentation on its reproductive biology (USDA Conservation Stewardship Program 2023). *Eryngium yuccifolium* is promoted by the USDA‐NRCS CSP for its value in supporting pollinators and other beneficial insects and has also been recommended to mitigate soil erosion (USDA Conservation Stewardship Program 2023; Berry et al. 2024). This species serves as a host plant for *Papaipema eryngii* Bird, 1917: Noctuidae, a moth that depends on *Eryngium yuccifolium* to complete its life cycle (Berry et al. 2024; Molano‐Flores et al. 2024). *Eryngium yuccifolium* is thought to be xenogamous, with partial self‐compatibility (Molano‐Flores 2001). *Gaillardia pulchella* is recommended by the USDA‐NRCS CSP to provide pollinator and beneficial insect habitat, including use in upland wildlife plantings, highway rights‐of‐way revegetation, reclamation of energy exploration sites, and range seed mixes. Its reproductive biology remains poorly documented (Ballare et al. 2017; Maher and Reilley 2020; USDA Conservation Stewardship Program 2023). *Bidens laevis* has been characterized as having a high value for pollinators and has been used for erosion control and habitat recovery, yet its pollination biology is not fully understood (Qureshi and Anwar 2025). *Monarda punctata, V. alternifolia, V. virginica*, and *E. purpurea* have been recommended by the USDA‐NRCS CSP to provide pollinator and beneficial insect habitat, yet little is known about their reproductive biologies (USDA Conservation Stewardship Program 2023).

In 2019, eight wildflower species were planted (either from seed or transplanted as mature plants), whereas one species ( *B. laevis* ) occurred as a naturally growing population. The plant stage used for plot establishment was determined by the time required to achieve blooming in a single season. Therefore, five species likely to bloom their first year were germinated from seed sourced from Roundstone Seed LLC (Upton, Kentucky, USA): *D. illinoensis* , *D. floridanum*, *M. punctata* , *V. alternifolia* , and *V. virginica* . *Three* species unlikely to bloom the first year were sourced as 1‐*year‐old* potted plants from Superior Trees Inc. (Lee, Florida, USA) to ensure blooms: *E. purpurea* , *E. yuccifolium* , and *G. pulchella* . The five seeded species were pre‐treated according to specific biological requirements needed to break seed dormancy and induce germination. Seeds of *V. alternifolia* and *V. virginica* were cold moist stratified by placing seeds at 1.6°C for 60 days in separate 0.95 L Ziploc bags with moistened sand (Kirt 1996). Seeds of *D. illinoensis* and *D. floridanum* do not require cold stratification, yet need to be mechanically scarified. This was accomplished by placing the seeds of each species in separate 0.95 L Ziploc bags containing dry sand (Quikrete Premium Play Sand (Atlanta, Georgia, USA)); each bag was then vigorously shaken for 5 min (Kimura and Islam 2012). *Monarda punctata* seeds require no pre‐treatment prior to planting (Anderson 2003) and were planted directly into seed starter trays (Gardner 2011).

In February 2019, once seeds of all species were pretreated as necessary, two seeds per species were planted (placed on the soil surface and not covered) in a single cell within a 72‐cell seed starter tray filled with dampened potting soil (Scotts Miracle‐Gro, Marysville, OH, USA). Seedlings were kept indoors and exposed to 16 h of light per day via fluorescent full‐spectrum (6000 K) grow lights (Bubel and Nick 2018). The soil surface was allowed to dry between waterings to prevent fungal and bacterial growth, and trays were bottom‐watered to limit damage to the seedlings (Bubel and Nick 2018). Once each seedling produced the first set of true leaves, the weakest of the two seedlings (if both seeds germinated) was thinned by clipping the stem at the soil level (Bubel and Nick 2018). Beginning on 16 April 2019, all seedlings were placed outside under the shade of a tree and were hardened off over two weeks (Stephens 2022). To harden off, seedlings were brought outdoors, and the time spent outdoors was increased each consecutive day by 30 min (starting with an hour left outdoors in the early morning at 08:00). Additionally, the seedlings were simultaneously moved from an increasing spectrum of full shade to full sun until they could be left outdoors for the entire day (Stephens 2022). All plants were monitored daily and were top‐watered with a mister (Orbit Max 8‐Pattern Nozzle, San Francisco, CA, USA) as needed.

### 2.2 Plot Preparation

**FIGURE 1:**
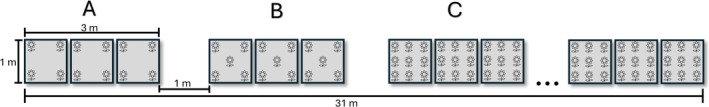
Layout of eight wildflower species planted at the Auburn University Bee Lab, Auburn, AL, USA, in 2019. Each species was planted in a 1 m^2^ subplot with three replicates. Each species was separated by a 1 m^2^ buffer. For clarity, only three of the eight plots are shown, with a fourth indicating plot continuation. Planting density (4, 5, or 9 plants per m^2^, labeled A, B, and C) was based on mature plant size to achieve nearly full vegetation cover. *Monarda punctata* and *Eryngium yuccifolium* were planted in arrangement A, *Gaillardia pulchella* , *Verbesina alternifolia* , and *Verbesina virginica* in B, and *Echinacea purpurea* , *Desmanthus illinoensis* , and *Desmodium floridanum* in C. Plant density for *Bidens laevis* was not quantified as the plants chosen for the study were a natural growing population alongside a pond bank at the Agricultural Heritage Park in Auburn, Alabama, which made quantifying the number of plants per square meter too difficult.

Two weeks before planting, a 10% concentration of glyphosate (Roundup, Monsanto, St. Louis, MO, USA) was sprayed over a 2 m × 41 m area at the Auburn University Bee Lab (32° 35.929'N, 85° 30.101'W) using a backpack sprayer to reduce weed competition and was rototilled two weeks later (Norcini and Aldrich 2004). On 30 April 2019, all 8 wildflower species (both started from seed and mature potted plants) were individually transplanted into three adjacent 1 × 1 m plots, forming a single 3 × 1 m strip per species. Each 3 × 1 m species‐specific strip was separated from the next by a 1 m^2^ buffer (Figure 1). The number of plants per species planted per 1 m^2^ plot—either 4, 5, or 9 individuals—was selected to achieve near 100% vegetation cover while minimizing crowding. Planting densities were based on the mature size of each species (Garbuzov and Ratnieks 2014) and recommendations from Superior Trees Inc. and Roundstone Seed LLC (Table 1 and Figure 1). Lastly, one wildflower species, *B. laevis* , was observed growing naturally on Auburn University's campus and was utilized for this study. This population was growing along a pond edge at Auburn University's Agricultural Heritage Park (32° 35.710'N, 85° 29.456'W) from 24 September to 7 November 2019. To maintain consistency across all wildflower species, we designated one 3 m^2^ plot (3 × 1 m dimensions) for *B. laevis* to focus our data collection.

**TABLE 1:** Plant families and scientific names of the nine native wildflower species used in insect exclusion experiments and pollinator surveys conducted in Auburn, Alabama (2019).

Family	Wildflower species	Fruit type	Bloom duration	Plants/1 m^2^	*N*	Sampling days	Number of inflorescences per treatment		Number of plants used for the exclusion study	Seed counts per inflorescence (open vs. bagged)	Weight per seed per inflorescence (open vs. bagged)
							open	bagged			
Apiaceae	*Eryngium yuccifolium* Michx.	Schizocarp	June–Aug	4	28	12	44	34	6	0	+
Asteraceae	*Bidens laevis* (L.) Britton, Sterns, & Poggenb.	Capsela	Sept–Nov	NA	60	14	25	18	NA	+	+
	*Echinacea purpurea* (L.) Moench	Capsela	May–Oct	9	59	29	19	23	10	+	+
	*Gaillardia pulchella* Foug.	Capsela	May–Aug	5	35	15	32	40	17	0	+
	*Verbesina alternifolia* Britton ex Kearney (L.)	Capsela	Aug–Oct	5	51	20	24	22	6	0	+
	*Verbesina virginica* L.	Capsela	May–Sept	5	20	14	37	24	NA	+	+
Fabaceae	*Desmanthus illinoensis* (Michx.) MacMill. ex B. L. Rob. & Fernald	Schizocarp	July–Sept	9	14	14	40	32	12	+	—
	*Desmodium floridanum* Chapm.	Legume	Sept–Oct	9	12	8	NA	NA	NA	NA	NA
Lamiaceae	*Monarda punctata* L.	Nutlet	Aug–Oct	4	29	12	24	22	6	+	0

*Note:* *N* indicates the total number of 10‐min sweep netting events conducted during each species' bloom period, and “sampling days” refers to the number of days these events occurred. Planting density per 1 m^2^ was selected based on each species' mature growth habit to achieve full vegetation cover. *Bidens laevis* was part of a naturally occurring population with individual plants entangled; due to the difficulty of accurately quantifying each plant, its planting density was not manipulated nor quantified (NA). In the “Seed Counts per inflorescence” and “Weight per Seed per Inflorescence” columns, symbols represent statistical differences between open‐pollinated and bagged inflorescences: 0 = no difference, + = significant increase, − = significant decrease. Seed analyses were not conducted for *Desmodium floridanum* due to insufficient seed production (NA).

### 2.3 Insect Exclusion Study

To determine the degree of insect pollinator dependency for each wildflower species, we conducted an insect exclusion experiment, whereby we completely enclosed unopened inflorescences prior to blooming (Insect Excluded) using a 4.5 L fine mesh (150 μm) polyester pail strainer (The Cary Company, Addison, IL, USA) (Kearns and Inouye 1993; McIver and Erickson 2012). Bagging the inflorescence prevents all insect pollinators except for the smallest biotic pollinators (e.g., thrips) from accessing the inflorescences, yet does not prevent the passive passage of pollen via wind through the bag (Neal and Anderson 2004). We also tagged, but did not bag, unopened inflorescences (open‐pollinated) using red flagging tape during the bloom periods of each wildflower species. Flagging, but not bagging, the inflorescences of each wildflower species enables insects to visit the inflorescences and transfer pollen freely. For all species that exhibit composite flowers (*B. laevis, D. illinoensis*, *V. alternifolia* , *V. virginica* , *E. purpurea* , *E. yuccifolium* , and *G. pulchella* ), we counted the entire composite inflorescence as a single flowering unit, and we further defined a single flowering unit for *D. floridanum* as the collection of flowers in bloom on a single panicle, and the apical 3 glomerules of a single branch for *M. punctata* (Smith et al. 2024).

Experimental treatments were applied evenly among the plants of each species to account for variation at the plant level (i.e., each plant received both the insect‐excluded and open‐pollinated treatments). All bags and flagging tape remained on the inflorescences during the duration of each wildflower species' bloom period. For each wildflower species and treatment (insect‐excluded and open‐pollinated flowers), once a seedhead formed, matured, and senesced, it was collected, placed in an individual brown paper bag, and allowed to dry naturally in indoor conditions (Blaauw and Isaacs 2014). When each seed head was completely dried, it was opened so that the number of mature seeds could be counted and weighed (Blaauw and Isaacs 2014). All mature seeds were counted and weighed to the nearest thousandth of a milligram with a digital scale (VWR‐124B, Randor, Pennsylvania, USA). Allocating inflorescences to insect‐excluded (i.e., bagged) and open‐pollinated (i.e., open) treatments allowed for comparisons between the number of seeds produced per inflorescence per treatment for each wildflower species. Furthermore, we compared the mean weight per seed per inflorescence for each treatment and wildflower species (Elisante et al. 2020); only inflorescences that produced one or more seeds were included in the seed weight analysis.

The number of seed heads analyzed per wildflower species varied depending on plant production (the number of inflorescences produced per plant) and bloom duration. The number of inflorescences included for the seed count analysis per wildflower species was: *B. laevis* (18:25:NA) (insect‐excluded inflorescences: open‐pollinated inflorescences: total plants), *D. illinoensis* (32:40:12), *E. purpurea* (23:19:10), *E. yuccifolium* (44:34:6), *G. pulchella* (40:32:17), *M. punctata* (22:24:6), *V. alternifolia* (203:253:5), and *V. virginica* (24:37:NA) (Table 1). For *B. laevis*, plant count was not recorded due to the difficulty assessing individual plants in the dense growth, and *V. virginica* plants were not recorded as the plant information on the labels had been damaged, though a total of 15 plants occurred within the 3 × 1 m species‐specific strip. Additionally, the number of seed heads utilized for the seed weight analysis per wildflower species differed from the number of inflorescences utilized for the seed count analysis because not all inflorescences produced seeds. The number of inflorescences utilized for the seed weight analysis per wildflower species was (insect‐excluded: open‐pollinated): *B. laevis* (15:25), *D. illinoensis* (10:40), *E. purpurea* (23:19), *E. yuccifolium* (44:32), *G. pulchella* (38:31), *M. punctata* (21:24), *V. alternifolia* (177:252), and *V. virginica* (22:37) (Table 1). This equated to three inflorescences failing to produce seeds in the insect‐excluded treatment of *B. laevis*, 22 in the insect‐excluded treatment of *D. illinoensis* , 2 in the open treatment of *E. yuccifolium*, 3 and 1 in the insect‐excluded and open treatments of *G. pulchella* , respectively, 26 and 1 in the insect‐excluded and open‐pollinated treatments of *V. alternifolia* , respectively, and 2 in the insect‐excluded treatment of *V. virginica*.

### 2.4 Floral Insect Visitor Survey

**FIGURE 2:**
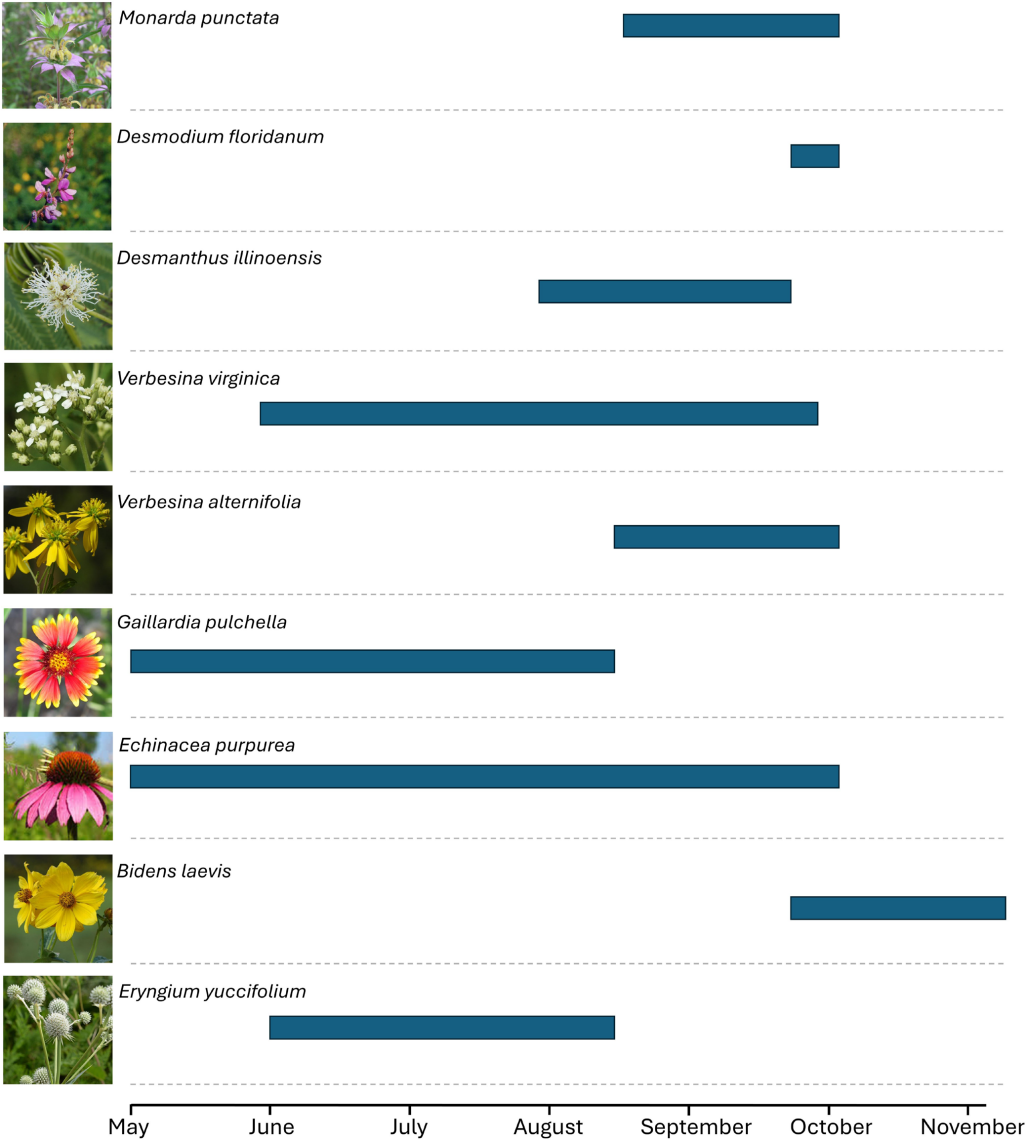
Bloom phenology for each wildflower species assessed in this study. The x‐axis represents the month of the year, and the y‐axis represents each wildflower species. The blue polygons represent each species' bloom period. The polygons correspond to the dates each species was netted for pollinators (i.e., in bloom) during the study. Photo credit: *Bidens laevis, Gaillardia pulchella*, and *Verbesina alternifolia—*Larry Allain, USGS*; Eryngium yuccifolium—*Christopher David Benda, USDA Forest Service; *Desmodium floridanum—*Jennifer Anderson, USDA‐NRCS PLANTS Database; *Echinacea purpurea—*no author specified, *USDA NRCS; Verbesina virginica* and *Desmanthus illinoensis—*A. P. Abbate.

We conducted timed, targeted sweep netting surveys concurrently with the insect exclusion study to determine the common floral insect visitors for all nine wildflower species. To maximize insect capture and to standardize sampling procedures, surveys were conducted over multiple days and sampling times (morning, midday, and afternoon) (Prado et al. 2017), and when weather conditions were appropriate for sampling foraging insects—warm (> 20°C) sunny days (< 60% cloud cover) with ≤ 40% chance of precipitation (Hopwood 2008). For each wildflower species, any plot containing at least one inflorescence in bloom was monitored for 10 min (Erickson, Grozinger, and Patch 2022; Erickson, Junker, et al. 2022). The number of sweep netting events varied for each wildflower species, reflecting the differences in each species' bloom duration throughout the year (Table 1 and Figure 2). For *B. laevis*, sweep netting surveys were restricted to a 3 × 1 m plot so the area swept was comparable in size to the planted wildflower plots at the Auburn University Bee Lab. Any insect pollinator observed visiting the inflorescences of a wildflower species was captured with a sweep net and stored in a 5 mL vial. After each 10‐min survey, insect pollinators were either identified in the field, recorded, and released, or placed in a cooler with ice and then identified in the laboratory. All insect pollinators were identified to the lowest taxonomic rank with the use of taxonomic keys (Bohart et al. 1976; McAlpine et al. 1981; Arnett Jr and Thomas 2000; Arnett Jr et al. 2002; Buck et al. 2008; Ascher and Pickering 2020). If insect pollinators were observed visiting the inflorescences but could not be captured, they were recorded at the genus level if possible.

### 2.5 Statistical Analyses

#### 2.5.1 Seed Count and Weight Assessments

To elucidate patterns in seed production across the wildflower species, we constructed and compared multiple generalized linear mixed models (GLMMs) fitted with the glmmTMB() function from the glmmTMB package in R (Brooks et al. 2017). We first constructed models to assess seed count and seed weight with treatment as a fixed effect and an interaction with plant species to include all wildflower species within a single model. We tested and compared models of varying combinations of fixed effects, random effects, and distribution families, yet we failed to identify an appropriate model as all models failed to converge, likely due to each flowering plant species exemplifying different data distributions. Instead, we opted to construct separate models for each wildflower species.

For each model, we constructed varying combinations of fixed effects, random effects, and distribution families to identify the best‐fitting model. We used scaled residual plots and quantile‐quantile plots generated with the DHARMa package (Hartig 2018) to assess model fit by visualizing zero‐inflation, under‐ or over‐dispersion, outliers, and to assess model fit. For all species, seed count was the response variable, treatment was the fixed effect, and a random intercept for bag number was included to account for variation among experimental units, except for *M. punctata* , where bag number was nested within plant number.

When multiple models showed adequate fit, we utilized Akaike's Information Criterion (AIC) to determine the best‐fitting model (see Table S1). For *D. illinoensis* , *G. pulchella* , *B. laevis* , and *V. virginica* , a zero‐inflated negative binomial distribution was used to account for under‐ or overdispersion (Zuur et al. 2009). Post hoc pairwise comparisons of treatment effects with a Tukey's adjustment were performed using the emmeans() function from the emmeans package (Lenth 2022). Models for *V. alternifolia* and *E. yuccifolium* were assessed using case bootstrapping due to violations of model assumptions in GLMMs. For this, we first fit linear mixed‐effects models using the lmer function from the lme4 package (Bates et al. 2015) with treatment as a fixed effect and bag number as a random intercept. For both species, we utilized case‐resampling bootstrapping implemented with the ‘bootstrap’ function and *p*‐values were obtained using the ‘bootstrap_pvals’ function from the parameters package (Lüdecke et al. 2020) with 2000 iterations, resampling with replacement for each exclusion bag within a plant.

We followed this same procedure to assess differences in seed weight between the treatments for each wildflower species. For most species, we included bag number as a random effect to account for variation among sampling units, yet for *E. yuccifolium* and *G. pulchella* , an additional nested random effect for bag number nested within plant number was included as there was enough variation between plants. Seed weight was analyzed with either a Gaussian or Gamma distribution based on model diagnostics and fit (Zuur et al. 2009). A dispersion formula was included for *E. yuccifolium* to account for heteroskedasticity. Bootstrap methods as described above were implemented for *V. alternifolia* and *E. purpurea* due to violations of model assumptions of GLMMs.

#### 2.5.2 Insect Floral Visitor Abundance, Richness, Network

To elucidate patterns of insect floral visitor abundance and richness and insect floral visitor–flowering plant interactions among the flowering plant species, we constructed GLMMs and a network diagram. For insect floral visitor abundance and richness, we constructed and compared multiple GLMMs with varying fixed effects, random effects, dispersion parameters, and distribution families. We used scaled residual plots and quantile‐quantile plots generated with the DHARMa package (Hartig 2018) to assess model fit, and when multiple models showed adequate fit, we utilized Akaike's Information Criterion (AIC) to determine the best‐fitting model (see Table S1). Due to sparse floral insect visitors visiting the inflorescences of *D. illinoensis* , *D. floridanum*, and *V. virginica* (22, 13, and 47 total individuals, respectively), the final model did not include these three species after all models failed to converge while including them singly and in combination.

The final model was a negative binomial GLMM, which included insect abundance as the response variable, plant species as the fixed effect, a dispersion formula by plant species, and an offset for the number of targeted sweep netting surveys completed per plant species to account for varying levels of sampling intensity among the wildflower species as each exemplified different lengths of bloom time (Abbate et al. 2024). Post hoc comparisons with a Tukey's adjustment were performed using the emmeans() function from the emmeans package to elucidate differences in insect floral visitor abundance among the wildflower species. We followed the same model‐building procedure and post hoc comparisons for insect floral visitor richness with the same model structure as insect floral visitor abundance.

To visualize and quantify plant‐pollinator interactions, we constructed a bipartite network using insect visitation data collected from the sweep net surveys using the function plotweb() in the bipartite package (Dormann et al. 2008), and calculated network‐level and species‐level metrics using networklevel() and specieslevel() functions, respectively. All statistical analyses were performed in R version 4.4.1 (R Core Team 2024). All visualizations were facilitated through the tidyverse packages (Wickham et al. 2019).

## 3 Results

### 3.1 Insect Exclusion Study

**FIGURE 3:**
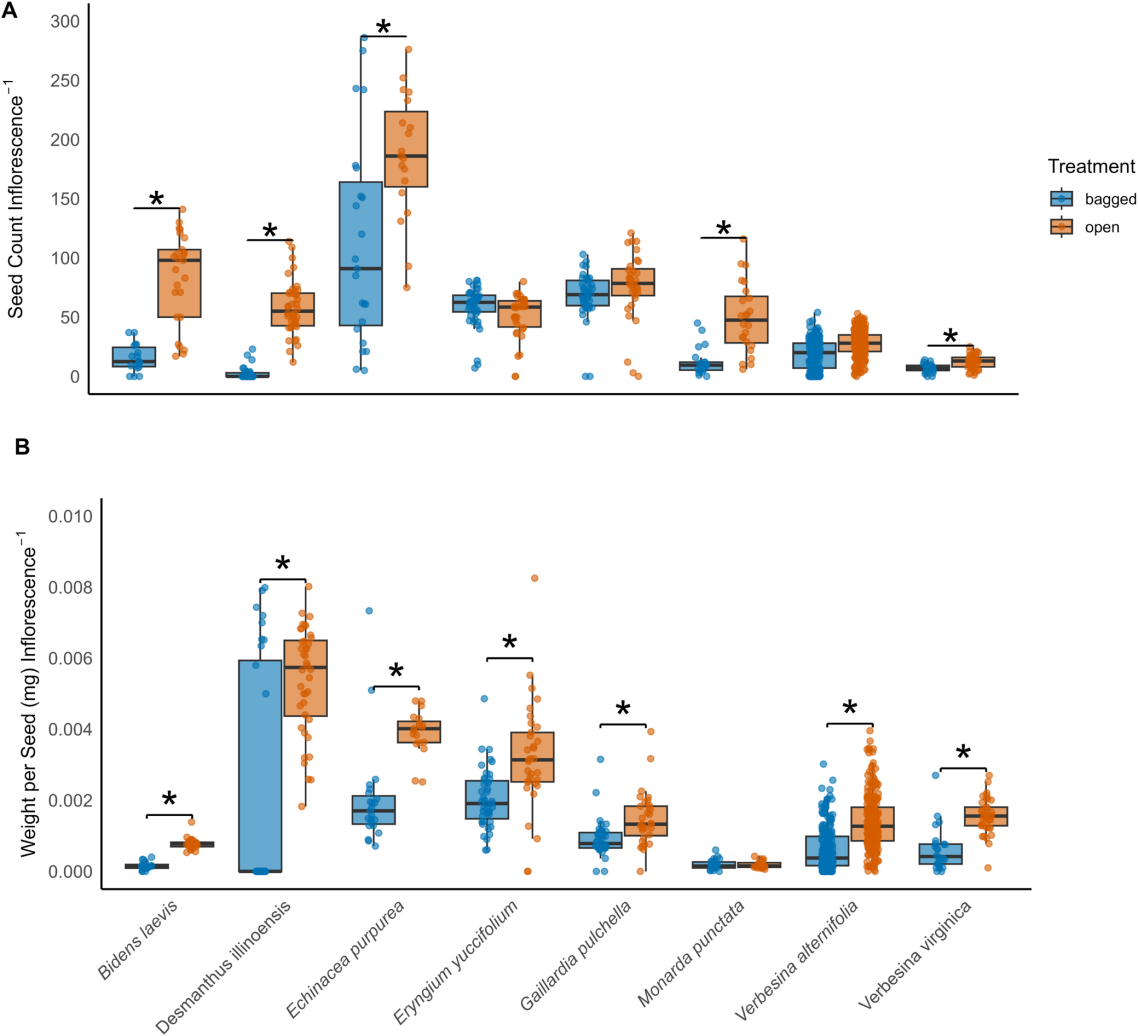
(A) Seed count per inflorescence and (B) seed weight per inflorescence for open (i.e., open‐pollinated)‐ (orange) and bagged (i.e., insect‐excluded) (blue) treatments across eight wildflower species. Boxplots display the median (line), interquartile range (box), and 1.5× interquartile range (whiskers); individual data points are overlaid with a jitter. Asterisks indicate significant differences in mean seed counts per inflorescence or mean seed weights per inflorescence per species between treatments (α = 0.05).

A total of 455 insect‐excluded and 453 open‐pollinated inflorescences were evaluated across all wildflower species from April to October 2019. Excluding insects from the inflorescences had a significant effect on the mean number of seeds produced per inflorescence in multiple plant species. Open‐pollinated inflorescences of *B. laevis* produced 71.2 ± 9.94 (estimated marginal mean ± SE) seeds per inflorescence, compared to 17.8 ± 3.41 in the insect‐excluded treatment, resulting in 4.00 times as many seeds in the open treatment (2.53–6.32: LCL‐UCL, *p* < 0.001; Negative Binomial GLMM) (Figure 3A and Table 1); *D. illinoensis* produced 57.9 ± 4.02 seeds in the open‐pollinated treatment and 8.55 ± 1.47 in the insect‐excluded, a 6.76‐fold difference (4.7–9.73: LCL‐UCL, *p* < 0.001; Negative Binomial GLMM)*; E. purpurea* produced 172 ± 21.4 seeds when open‐pollinated and 106 ± 22.4 when bagged, a 1.62‐fold difference (1.12–2.35: LCL‐UCL, *p* = 0.009; Negative Binomial GLMM); *M. punctata* produced 49.4 ± 11.3 seeds per inflorescence in the open‐pollinated treatment and 11.7 ± 2.37 in the bagged, a 4.22‐fold difference (2.38–7.48: LCL‐UCL, *p* < 0.0001; Negative Binomial GLMM); *V. virginica* produced 12.42 ± 1.49 in the open‐pollinated treatment compared to 7.52 ± 0.818 in the insect‐excluded treatment, a 1.65‐fold difference (1.2–2.27: LCL‐UCL, *p* = 0.002; Negative Binomial GLMM). In contrast, several species exhibited no statistical differences in seed production between the treatments. *Eryngium yuccifolium* produced 59.1 ± 8.76 seeds per inflorescence when open‐pollinated and 45.5 ± 7.67, a 1.3‐fold difference in seed production between the treatments (0.81–2.05: LCL‐UCL, *p* = 0.41; LMM case bootstrap); *G. pulchella* produced 77.7 ± 6.46 seeds per inflorescence in the open‐pollinated treatment and 71.7 ± 2.30 when bagged, a 1.08 times difference between treatments (0.91–1.29: LCL‐UCL, *p* = 0.37; Negative Binomial GLM), and *V. alternifolia* produced 25.2 ± 1.88 seeds in the open‐pollinated treatment and 21.0 ± 1.53 in the insect‐excluded treatment, a 1.21 times difference between treatments (0.97–1.49: LCL‐UCL, *p* = 0.059; LMM case bootstrap), though marginally non‐statistically different (Figure 3A).

Mean weights per seed per inflorescence were significantly lower in insect‐excluded treatments compared to open‐pollinated treatments for multiple species. In *B. laevis*, open‐pollinated inflorescences produced seeds that were heavier (7.78 ± 0.33) × 10^−4^ mg compared to bagged inflorescences (1.83 ± 0.49) × 10^−4^ mg, a difference of 5.97 × 10^−4^ mg (4.97–6.96 × 10^−4^: LCL‐UCL, *p* < 0.0001; Gaussian GLMM) (Figure 3B and Table 1). Seeds from open‐pollinated inflorescences of *E. purpurea* were heavier (3.86 ± 0.31) × 10^−3^ mg compared to bagged inflorescences (2.11 ± 0.28) × 10^−3^ mg, a difference of 1.75 × 10^−3^ mg (9.12–2.59 × 10^−3^: LCL‐UCL, *p* < 0.0001; Gaussian GLMM). Seeds from open‐pollinated inflorescences of *V. virginica* were (1.47 ± 0.23) × 10^−3^ mg, compared to (7.49 ± 1.78) × 10^−4^ mg in bagged inflorescences, a 7.00 × 10^−4^ mg difference (1.38–1.30 × 10^−3^: LCL‐UCL, *p* = 0.017; Gaussian GLMM). Seeds from open‐pollinated inflorescences of *V. alternifolia* were significantly heavier (1.44 ± 0.15) × 10^−3^ mg than in the insect‐excluded treatment (5.26 ± 1.20) × 10^−4^ mg, with a difference of 9.2 × 10^−4^ mg (5.5–13.1 × 10^−4^: LCL‐UCL, *p* = 0.0005; LMM case bootstrap). Seeds from open‐pollinated inflorescences of *E. yuccifolium* were (3.82 ± 0.41) × 10^−3^ mg and (2.18 ± 0.25) × 10^−3^ mg in the insect‐excluded treatment, resulting in open‐pollinated seeds being 0.571 times heavier (0.415–0.784: LCL‐UCL, *p* = 0.0005; Gamma Log GLMM). Seeds from open‐pollinated inflorescences of *G. pulchella* were (1.48 ± 0.13) × 10^−3^ mg vs. (9.59 ± 0.86) × 10^−4^ mg when bagged, 1.55 times heavier than those from insect‐excluded inflorescences (1.21–1.99: LCL‐UCL, *p* = 0.0006: Gamma Log GLMM). Conversely, seeds from open‐pollinated inflorescences of *D. illinoensis* showed the opposite trend, with insect‐excluded inflorescences producing heavier seeds (6.77 ± 0.44) × 10^−3^ mg than open‐pollinated inflorescences (5.39 ± 0.22) × 10^−3^ mg, a difference of 1.39 × 10^−3^ mg lighter than those from insect‐excluded inflorescences (−2.38 to −4.00 × 10^−3^: LCL‐UCL, *p* = 0.0071; Gaussian GLMM). Lastly, seeds from open‐pollinated inflorescences of *M. punctata* showed no significant differences in seed weight between treatments, with open‐pollinated seeds weighing (1.85 ± 0.30) × 10^−4^ mg and seeds derived from insect‐excluded inflorescences weighing (2.07 ± 0.31) × 10^−4^ mg, a 2.0 × 10^−5^ mg difference between treatments (−1.11–0.067 × 10^−4^: LCL‐UCL, *p* = 0.625; Gaussian GLMM) (Figure 3B).

### 3.2 Floral Visitor Identification Survey

A total of 308 10‐min targeted sweep‐netting surveys were conducted across the 9 wildflower species from 9 May 2019 to 7 November 2019. The total number of 10‐min targeted sweep‐netting events per wildflower species is as follows and was dependent upon each species' bloom duration: *B. laevis* (60), *D. illinoensis* (14), *D. floridanum* (12), *E. purpurea* (59), *E. yuccifolium* (28), *G. pulchella* (35), *M. punctata* (29), *V. alternifolia* (51), *V. virginica* (20) (Table 1). Through the targeted sweep‐netting events, a total of 1417 insects, representing 73 genera/species from 21 families and 4 orders, were observed visiting the 9 wildflower species (Table 2). Most floral visits were by bees (66.2%), followed by wasps (14.5%), butterflies and moths (7.1%), flies (6.1%), and beetles (6.1%).

**TABLE 2:** List of insect pollinator order, family, genus/species, and their abundance while visiting the 9 planted wildflower species from April‐October 2019 in Auburn, Alabama. Total pollinator visits, pollinator richness, and the percentage of insect pollinator visits are listed at the bottom of the table.

	*Bidens laevis*	*Desmanthus illinoensis*	*Desmodium floridanum*	*Echinacea purpurea*	*Eryngium yuccifolium*	*Gaillardia pulchella*	*Monarda punctata*	*Verbesina alternifolia*	*Verbesina virginica*	Total
**Coleoptera**										
**Cantharidae**										
*Chauliognathus* sp. Hentz, 1830									1	1
**Chrysomelidae**										
*Diabrotica* sp. Chevrolat in Dejean, 1836							1	1		2
**Mordellidae**										
*Euphoria sepulcralis* Fabricius, 1801									1	1
*Mordella* sp. L. 1758					12	56			5	73
**Scarabaeidae**										
*Trigonopeltastes delta* Foster, 1771				4	5					9
**Bibionidae**										
*Plecia nearctica* Hardy, 1940									7	7
**Calliphoridae**										
*Lucilia* sp. Robineau‐Desvoidy, 1830	1			1						2
**Conopidae**										
*Physocephala* sp. Schiner, 1861						1		1		2
**Syrphidae**										
*Allograpta obliqua* Say, 1823	1									1
*Dioprosopa clavata* Hull, 1949		2								2
*Eristalis dimidiata* (Wiedemann, 1830)	25									25
*Eristalis* sp. Latreille, 1804	5									5
*Eristalis transversa* (Wiedemann, 1830)	15									15
*Eupeodes* sp. Osten Sacken, 1877	5									5
*Helophilus fasciatus* Walker, 1849	9									9
*Ocyptamus fuscipennis* Macquart, 1834							1			1
*Palpada furcata* Wiedemann, 1819	1				1					2
*Syritta flaviventris* Macquart, 1842					1	1				2
*Toxomerus* sp. Macquart, 1855	2			3						5
**Tachinidae**										
*Archytas* sp. Jaennicke, 1867								1		1
**Hymenoptera (Bees)**										
**Andrenidae**										
*Andrena accepta* Viereck, 1916	1									1
**Apidae**										
*Apis mellifera* L. 1758	94		4	193	10	17	17	41	8	384
*Bombus impatiens* Cresson, 1863	124			1				1		126
*Bombus* sp. Latreille, 1802				1						1
*Melissodes bimaculatus* Lepeletier, 1825				1						1
*Melissodes communis* Cresson, 1878								1		1
*Melissodes comptoides* Robertson, 1898						1				1
*Xylocopa virginica* L. 1771	8	1	1	3		1	16	4		34
**Halictidae**										
*Augochlora metallica* (Fabricius, 1793)	3									3
*Halictus parallelus* Say, 1837	1									1
*Halictus poeyi/ligatus* Smith, 1853/Say, 1837	5	1	1	22		245		34	19	327
*Lasioglossum callidum* (Sandhouse, 1924)					1		4			5
*Lasioglossum floridanum* (Robertson, 1892)				3						3
*Lasioglossum imitatum* (Smith, 1853)		17			4	5	1			27
*Lasioglossum leviense/weemsi/mitchelli*		1								1
*Lasioglossum longifrons* (Baker, 1906)					1	1				2
*Lasioglossum* spp. (Curtis, 1833)	1		3	4	1	5				14
**Megachilidae**										
*Megachile integra* Cresson, 1878	1									1
*Megachile* sp. Latreille, 1802				1		4				5
**Hymenoptera (Wasps)**										0
**Chalcididae**			1							1
**Crabronidae**										
*Bicyrtes* sp. Lepeletier, 1845					1					1
*Cerceris bicornuta* Guérin‐Méneville, 1844					3		6			9
*Philanthus* sp. Fabricius, 1790					1					1
**Scoliidae**										
*Scolia bicincta* Fabricius, 1775							3			3
*Scolia dubia* Say, 1837								19		19
*Scolia nobilitata* Fabricius, 1805				6	75		3	26	3	113
**Sphecidae**										
*Ammophila fernaldi* (Murray, 1938)					1					1
*Ammophila procera* Dahlbom, 1843					1		2	4		7
*Ammophila* sp. W. Kirby, 1798			1							1
*Eremnophila aureonotata* (Cameron, 1888)	1						7	2		10
*Sphex dorsalis* Lepeletier de Saint Fargeau, 1845							5			5
*Sphex flavovestitus* F. Smith, 1856			1				2			3
*Sphex ichneumoneus* (Linnaeus, 1758)					3		6	4		13
*Sphex pensylvanicus* Linnaeus, 1763							3			3
**Vespidae**										
*Eumenes fraternus* Say, 1824	1								1	2
*Euodynerus crypticus* (Say, 1823)							1			1
*Monobia quadridens* (Linnaeus, 1763)							4			4
*Polistes carolina* (Linnaeus, 1767)			1							1
*Pseudodynerus quadrisectus* Say, 1837							3			3
*Vespula germanica* (Fabricius, 1793)	1									1
*Vespula squamosa* (Drury, 1773)	1									1
*Zethus spinipes* Say, 1837							1	2	2	5
**Lepidoptera**										
**Attevidae**										
*Atteva aurea* (Fitch, 1856)				1						1
**Hesperiidae**	1									1
*Atalopedes campestris* Boisduval, 1852				2						2
*Copaeodes minima* (W.H. Edwards, 1870)				2	1			1		4
*Hylephila phyleus* (Drury, 1773)	1			44		7		1		53
*Lerodea eufala* (Edwards, 1869)				1						1
*Panoquina ocola* (W.H. Edwards, 1863)				1						1
*Pyrgus albescens* Grishin, 2022				3						3
*Pyrgus* sp. Hübner, [1819]				1	2					3
**Nymphalidae**										
*Danaus plexippus* (Linnaeus, 1758)	18									18
*Junonia coenia* Hübner, 1822				5		1		3		9
*Vanessa cardui* (Linnaeus, 1758)				3		1				4
*Vanessa virginiensis* (Drury, 1773)				1						1
Total Pollinator Abundance	326	22	13	307	124	346	86	146	47	**1417**
Pollinator Richness	25	5	8	24	18	14	19	17	9	**76**
Percentage of insect pollinator visits	23.0	1.6	0.9	21.7	8.8	24.4	6.1	10.3	3.3	

**FIGURE 4:**
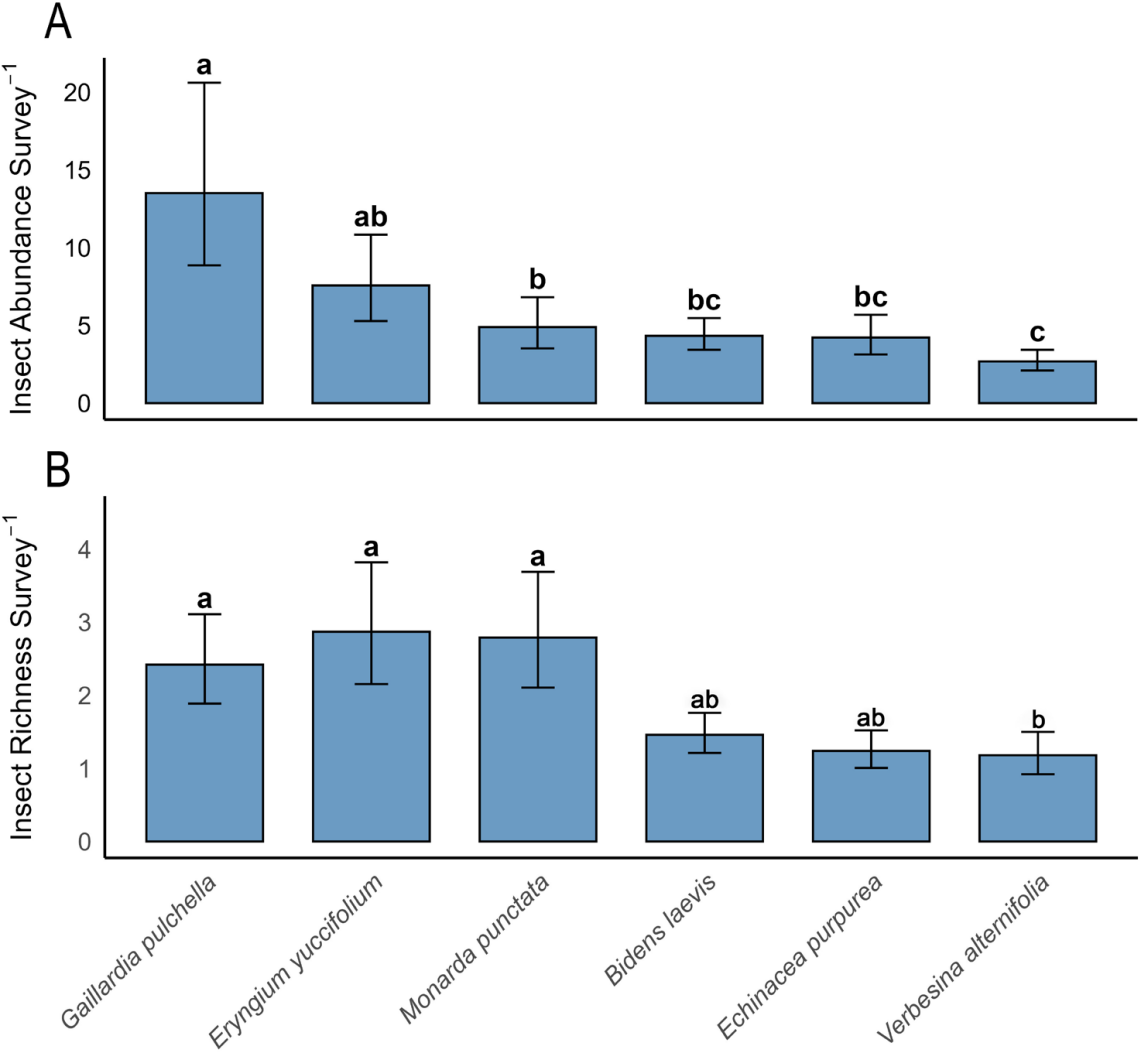
Estimated marginal means of insect floral visitor abundance and richness per sweep netting survey across six native wildflower species. Confidence bands represent 95% confidence limits. *Verbesina virginica* , *Desmanthus illinoensis*, and *Desmodium floridanum* were excluded from analysis due to low insect floral visitor abundances. (A) Insect abundance per survey was modeled using a negative binomial GLMM with the number of surveys conducted per species as an offset to account for variation in sampling intensity. (B) Insect richness per survey was modeled using a negative binomial GLMM, also including the number of surveys as an offset. Letters above bars denote statistically distinguishable groups based on Tukey‐adjusted pairwise comparisons (α = 0.05).

Three species of wildflowers (*D. floridanum, D. illinoensis*, and *V. virginica* ) were excluded from the analysis due to low insect visitor abundance (i.e., insufficient sample sizes for analysis). For the wildflower species included in the analysis, mean insect floral visitor abundance differed significantly among the wildflower species (Figure 4A). Estimated marginal means from the negative binomial GLMM indicated that *G. pulchella* attracted the highest bee abundance with 13.51 insect floral visitors per survey (8.86–20.61: LCL‐UCL), followed by *E. yuccifolium* (7.57; 5.28–10.83: LCL‐UCL), yet were not statistically different from each other (*p* = 0.31), followed by *M. punctata* (4.89; 3.52–6.81: LCL‐UCL), *B. laevis* (4.33; 3.43–5.47: LCL‐UCL), and *E. purpurea* (4.22; 3.13–5.68: LCL‐UCL) which were not statistically different from one another (all Ps > 0.10). Lastly, *V. alternifolia* attracted the fewest abundance of floral visitors per survey (2.68; 2.10–3.43; LCL‐UCL), and was not statistically different from *B. laevis* (*p* = 0.064) nor *E. purpurea* (*p* = 0.20).

Mean insect floral visitor richness differed significantly among the wildflower species (Figure 4B). Estimated marginal means from the negative binomial GLMM indicated that *E. yuccifolium* , *M. punctata*, and *G. pulchella* attracted the greatest richness of insect floral visitors compared to *B. laevis, E. purpurea*, and *V. alternifolia* (all Ps < 0.0025), yet were not statistically different from each other (all Ps > 0.05). *Bidens laevis, E. purpurea*, and *V. alternifolia* were not statistically different from one another (all Ps > 0.05) (Figure 4B).

**FIGURE 5:**
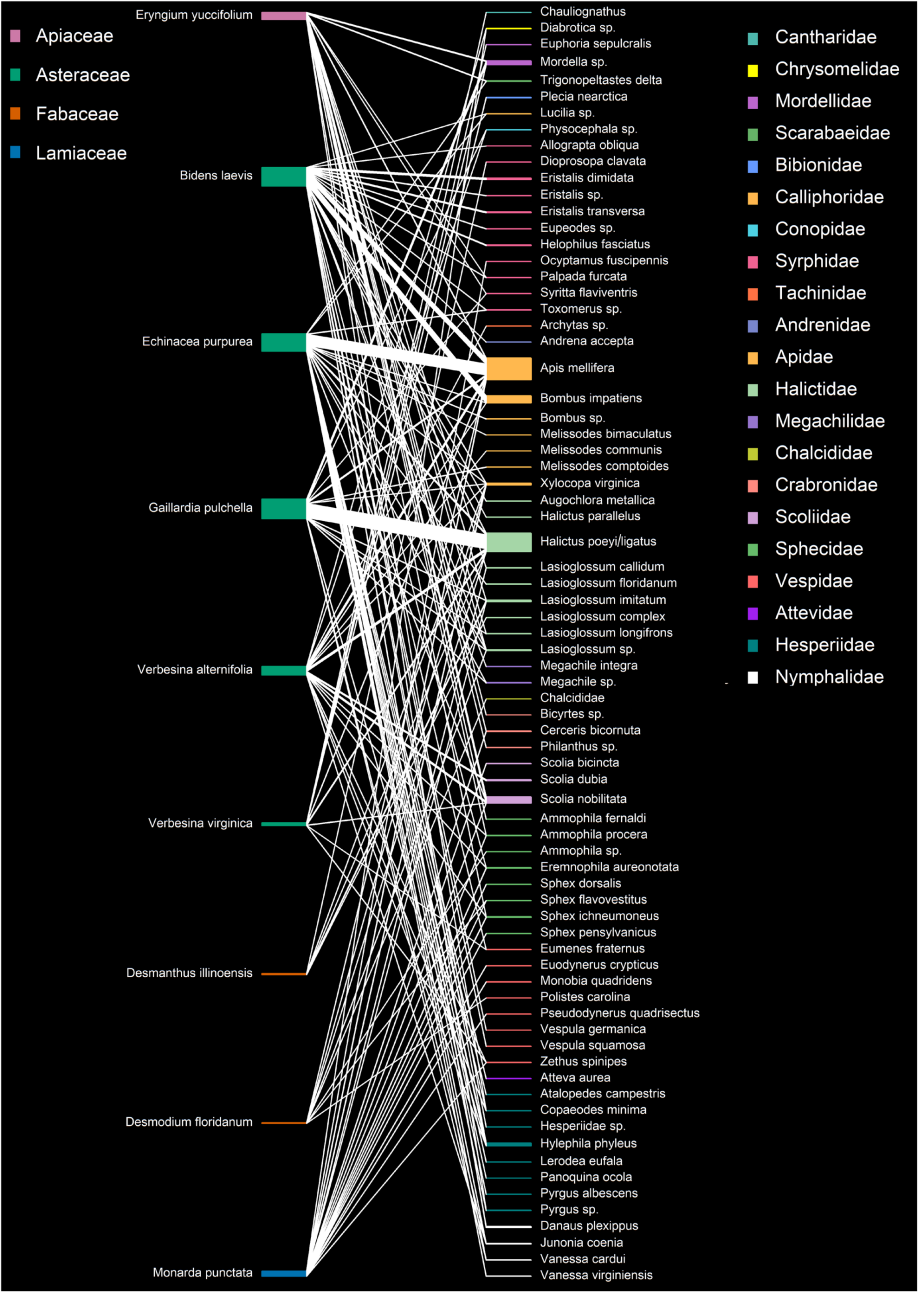
Bipartite network diagram of plant–pollinator interactions during standardized sweep net surveys. Each link represents the summed number of observed floral visits between pollinators (right nodes) and native wildflower species (left nodes) across all surveys conducted in the experiment. The width of each connecting line is proportional to the interaction frequency. The width of each colored polygon represents the relative abundance of each insect pollinator (right polygons) and the relative abundance of visits to the wildflower species (left polygons). Pollinator and plant species are grouped by family and color‐coded accordingly. Legends show family‐level color assignments and are positioned to the right (pollinators) and left (plants) of the web. The visualization was generated using the plotweb() function from the bipartite R package.

The network analysis revealed moderate connectance (0.21), high modularity (Q = 0.52), and moderate specialization (H2 = 0.55), reflecting compartmentalized interactions where many species show affinity for certain plant species, yet are not exclusive. More specifically, connectance is the proportion of the realized links out of all of the possible interactions between the wildflowers and insect pollinators. Modularity is a measure of how well the network is divided into distinct modules of tightly interacting species, with a high value reflecting compartmentalization. Specialization measures how exclusive interactions are across the entire network and highlights that many pollinators display preferences for specific wildflower species, though most are not entirely specialized. Despite a greater number of pollinators than plants (web asymmetry = 0.79), the network highlighted asymmetrical robustness: pollinators appeared more robust to species loss (robustness = 0.92) than plants (robustness = 0.49), indicating that if wildflower species are lost, insect pollinators can still visit other wildflower species (are more resilient). Conversely, the lower robustness value observed for the plants indicates that if pollinator species are lost, the plants are more vulnerable and may not get visited and pollinated. For the full network‐level and species‐level statistical outputs, see Tables S2 and S3. The network analysis also highlighted the three most common bee pollinators captured in this study, *Halictus poeyi/ligatus* Smith/Say (Halictidae), *Apis mellifera* L. (Apidae), and *Bombus impatiens* Cresson (Apidae). *Halictus poeyi/ligatus* was observed visiting 7 of the 9 wildflower species, with most floral visits to *G. pulchella* (75% of all visits) (Figure 5). *Apis mellifera* was observed visiting 8 of the 9 wildflower species with the most floral visits to *E. purpurea* (50% of all visits) and *B. laevis* (24% of all visits). Lastly, *B. impatiens* was observed visiting three wildflower species ( *B. laevis* , *E. purpurea* , and *V. alternifolia* ), with almost all floral visits to *B. laevis* (98%). The three most common wasps captured in this study were *Scolia nobilitata* Fabricius (Scoliidae), *Scolia dubia* Say (Scoliidae), and *Sphex ichneumoneus* L (Sphecidae). *Scolia nobilitata* Fabricius was observed visiting 5 of the 9 wildflower species, with most visits (89%) to *E. yuccifolium* and *V. virginica* . *Scolia dubia* Say was observed exclusively visiting the inflorescences of *V. alternifolia*, and *S. ichneumoneus* was observed visiting a combination of *E. yuccifolium* , *M. punctata* , and *V. alternifolia* with 23%, 46%, and 31% of their visits to each species, respectively.

Of the non‐hymenopteran insect visitors, *Mordella* (Mordellidae), Syrphidae, and *Hylephila phyleus* (Drury) (Hesperiidae) were the most observed non‐hymenopteran insect pollinators visiting the wildflowers in this study. *Mordella* were observed primarily visiting the inflorescences of *G. pulchella* (76% of all visits). Syrphid flies primarily visited inflorescences of *B. laevis* (88% of all visits). Of the syrphid flies, *Eristalis dimidiata* (Wiedemann), *Eristalis transversa* (Wiedemann), and *Helophilus fasciatus* (Walker) were the most abundant, and all their visits were to *B. laevis*. Lastly, *Hylephila phyleus* was the most abundant lepidopteran caught and primarily visited the inflorescences of *E. purpurea* (83% of all visits) (Figure 5).

## 4 Discussion

Through an insect exclusion experiment and targeted pollinator sweep netting surveys, we evaluated the reproductive dependence and pollinator associations of nine native wildflower species. Several species, including *B. laevis, E. purpurea, V. virginica, D. illinoensis*, and *M. punctata*, produced significantly more seeds and/or heavier seeds per inflorescence when insect pollinators were allowed to visit their inflorescences. This highlights a strong dependence on pollinators for reproductive success. In comparison, *E. yuccifolium, G. pulchella*, and *V. alternifolia* produced comparable seed counts per inflorescence in both open‐pollinated and bagged treatments, demonstrating less dependence on pollinators and a partial reliance on self‐pollination. In contrast, *E. yuccifolium* and *G. pulchella* produced heavier seeds when inflorescences were accessible to pollinators, indicating the contribution of insect pollinators to seed quality even when seed quantity is unaffected.

These patterns are consistent with studies that have demonstrated that insect pollinator visitation positively affects both the quantity and quality of seed production in various crops and wildflower species (Snow 1982; Steffan‐Dewenter et al. 2001; Bommarco et al. 2012). Seed reductions observed in our pollinator‐excluded treatments likely resulted from insufficient pollen deposition or low‐quality pollen (Wilcock and Neiland 2002; Aizen and Harder 2007). Many flowering crops and wildflower species often receive relatively low numbers of conspecific pollen grains and are sufficient for seed set and fruit set, yet the seeds and/or fruit can often be enhanced by larger numbers of deposited pollen grains (Snow 1982; Bommarco et al. 2012; Campbell et al. 2019; Stavert et al. 2020; Abbate et al. 2023). This has important implications for the fitness and persistence of a plant species, as smaller seeds, such as those produced under pollinator‐excluded conditions, tend to have lower viability, germination rates, and survival (Meagher et al. 1978; Banks 1980; Mehrhoff 1983; Mojonnier 1998; Walck et al. 2002; Miller et al. 2004; Dorsey and Wilson 2011; Combs et al. 2013), though this was not directly tested in our study and needs to be further examined. In this study, we were unable to quantify ovule number and thus the proportion of mature seeds per inflorescence to the total potential seeds. Combining absolute seed counts with seed weights provides complementary insights into reproductive success; future studies should consider recording potential seed set, as this would give an even more accurate indication of pollination success. Seeds with lower weights (i.e., smaller seeds) produced by insect‐excluded inflorescences may be less vigorous compared to seeds produced by open‐pollinated inflorescences (Ne'eman et al. 2010), yet this too needs to be examined further. Additionally, an important consideration is that individual seed weight may not consistently reflect pollination success, since plants often face a resource‐allocation trade‐off: higher seed set can dilute maternal resources per seed, reducing average seed weight even when pollination rates are high (Leishman 2001). However, for the majority of these species, both seed abundance and seed weight were positively associated with open‐pollination.

The variation in pollinator dependence among the wildflower species assessed in this study has practical implications for habitat restoration. Self‐compatible or partially self‐compatible species such as *E. yuccifolium* and *V. alternifolia* may be useful for conservation plantings when experiencing reduced pollinator activity due to degraded ecosystems or yearly fluctuations in insect pollinator abundance. Our results show that these species can reproduce and set seed in the absence of floral visitors while simultaneously providing floral resources for insect pollinators, highlighting self‐fertilization as a potentially beneficial trait for enhancing degraded habitats. In direct comparison, species showing strong pollinator dependence such as *B. laevis, E. purpurea*, and *V. virginica* could be ideal for inclusion in conservation plantings in areas that contain robust or recovering pollinator communities. Since these species have higher pollinator dependence, they are likely to experience reduced pollinator dependence pressure within robust or recovering pollinator communities, thus being able to reproduce and persist in the environment under these specific scenarios. Furthermore, including both pollinator‐dependent and self‐compatible wildflower species in a seed mix for conservation could reinforce plant‐pollinator interactions and increase redundancy while supporting higher abundance and diversity of insect pollinators.

Many of the wildflower species that had significant reductions in seed production and seed weights when excluded from insect pollinators ( *B. laevis* , *D. illinoensis, E. purpurea, M. punctata, V. alternifolia*, and *V. virginica* ) also attracted an abundance of insect pollinators. Due to floral morphology, bloom period, and floral rewards, each wildflower species attracted varying degrees of insect taxa that likely contributed to the pollination and production of viable seeds (i.e., wildflowers attracting a higher diversity of abundant pollinator species likely benefitted from increased outcrossing and greater pollinator redundancy) (Payne et al. 1989; Khan and Chaudory 1995; Stein et al. 2017). Generally, when flowering plants are exposed to a greater abundance and diversity of insect pollinators, they produce greater numbers of seeds and produce seeds of larger size and weight (Jennersten 1988; Atmowidi et al. 2007). An important caveat to note is that while higher pollinator abundance and species richness offer valuable aspects of insect pollinator communities, they may not always translate into greater reproductive success. For example, differences in foraging behavior and pollen transfer efficiency between insect pollinators mean that a community dominated by a few efficient pollinators can lead to higher seed set than one with many less efficient visitors (Wilcock and Neiland 2002). In contrast, the species *D. illinoensis, D. floridanum*, and *V. virginica* attracted relatively few pollinators. *Desmanthus illinoensis* produced heavier seeds in the absence of pollinators, suggesting wind pollination or self‐compatibility, which is consistent with previous observations (Latting 1961). The low seed production observed for *D. floridanum* could have been contributed to by its small inconspicuous inflorescences, late bloom period (July–October), production of few seeds per inflorescence (loment with 3–5 deltoid segments) (Weakley and Southeastern Flora Team 2024), unclear floral rewards (Miguel‐Peñaloza et al. 2019), and potential competition by neighboring wildflower species.

Our sweep netting surveys revealed that over 80% of the insect floral visitors were hymenopterans, including ecologically important bees and wasps. The most frequently observed bee, *Halictus poeyi/ligatus*, a floral generalist, was observed visiting 7 of the 9 flowering plant species, particularly *G. pulchella* . *Apis mellifera* visited 8 species, with most visits to *E. purpurea* and *B. laevis* . *Bombus impatiens*, also a floral generalist, is important in the pollination of crops and wildflower species (Shipp et al. 1994; Campbell et al. 2018; Abbate et al. 2023). *Bombus impatiens* visited only three species, with most visits to *B. laevis* , likely due to its late‐season bloom and abundant floral rewards when other floral resources were potentially scarce (Torres and Galetto 2002). Among the wasp species, *S. nobilitata* and *S. dubia* , parasitoids of soil‐inhibiting scarab beetle larvae, feed on nectar as adults (Abbate et al. 2018). These two species were the most common wasps, with most visits to *E. yuccifolium, V. virginica*, and *V. alternifolia* , which have shallow corollas suited for short mouthparts. *Sphex ichneumoneus* , a predator of orthopterans, has elongated mouthparts and is known to visit flowers for a nectar source. It was observed visiting a broad range of wildflower species, including *M. punctata, E. yuccifolium*, and *V. alternifolia*, potentially highlighting their ability to access nectar with flowers of varying morphologies (Coelho and Ladage 1999).

Of the non‐hymenopteran insect floral visitors, *Mordella* (Mordellidae), Syrphidae, and *Hylephila phyleus* (Hesperiidae) were the most common. *Mordella* beetles are regarded as important pollinators, readily visit flowers to feed on pollen (Liljeblad 1945; Ford and Jackman 1996; Bao et al. 2019), and were commonly observed visiting the inflorescences of *G. pulchella* . Several syrphid flies, including *Eristalis dimidiate* (Wiedemann), *Eristalis transversa* (Wiedemann), and *Helophilus fasciatus* (Walker) were the most common syrphids, with all visits to *B. laevis* likely due to its fall blooming period when other floral resources are limited (Clem et al. 2022). Lastly, the most common lepidopteran, *Hylephila phyleus* , primarily visited the inflorescences of *E. purpurea* , which produces large quantities of nectar (Clements 2012).

## 5 Conclusions

Although we documented the pollination requirements of eight, and the common floral visitors of nine native wildflower species native to the southeastern United States, the pollination requirements and breeding systems for thousands of native wildflower species remain largely unknown. The provision of floral resources to native pollinators is important to supporting their communities, and for some wildflowers, being visited by a high abundance and diversity of pollinators is equally important for their successful reproduction and persistence in the environment (Blaauw and Isaacs 2014). Our findings highlight the complexity of plant‐pollinator interactions and the importance of seed production and support for pollinators. Selecting native wildflower species that can offer both reproductive resilience and pollinator value in different ecological contexts is crucial. The information gained from this study can help shape native wildflower selections for conservation plantings and can be used to design effective seed mixes that can perform across a diversity of site conditions to support insect pollinators.

## Author Contributions

**Anthony P. Abbate:** writing – original draft (equal). **Joshua W. Campbell:** writing – original draft (equal). **Anthony W. Cuminale:** writing – original draft (equal). **Natalie M. West:** writing – original draft (equal). **Geoffrey R. Williams:** writing – original draft (equal).

## Conflicts of Interest

The authors declare no conflicts of interest.

## Supporting information


**Table S1:** ece372127‐sup‐0001‐Tables.xlsx.

## Data Availability

Data are freely available through Zenodo at https://doi.org/10.5281/zenodo.15360963.
